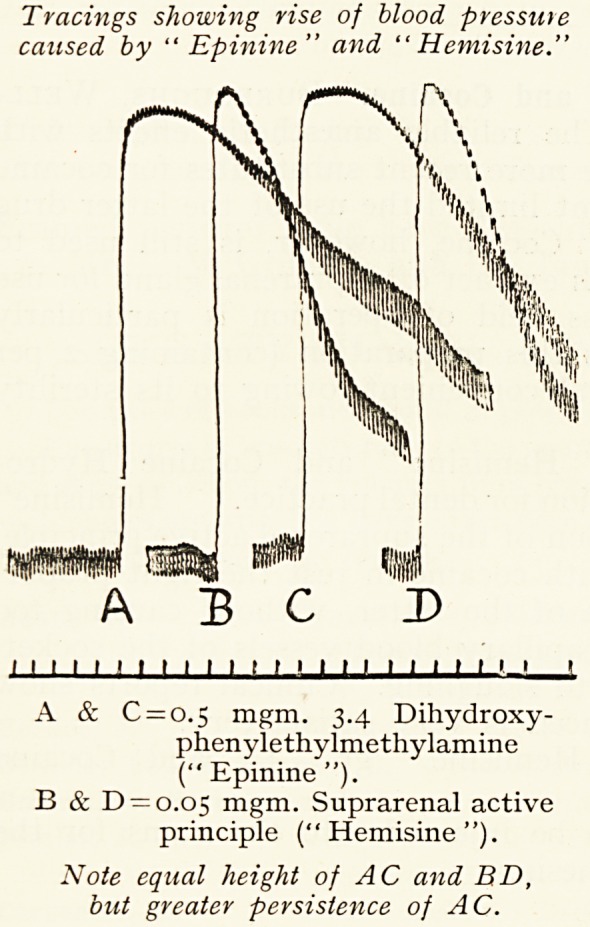# Notes on Preparations for the Sick

**Published:** 1911-03

**Authors:** 


### Notes on Preparations for the Sick.

**Streptococcus Vaccine, Rheumatic Fever.**—Burroughs, Wellcome & Co., London.—The number of diseases for the treatment of which stock vaccines are obtainable increases rapidly. The latest addition to the already lengthy list of vaccines issued by this firm is Streptococcus Vaccine, Rheumatic Fever. As is well known, the researches of Paine and Poynton and others have demonstrated the presence in rheumatic lesions and in the blood of rheumatic patients of an organism which is believed by some to be causally connected with rheumatic fever, and to which the name *micrococcus rheumaticus* has been given. It is from this organism that the new vaccine is obtained, several strains being used in its preparation. The vaccine is said to be useful in cases of persistent relapsing rheumatism. It is issued in convenient hermetically-sealed phials of 1 c.c., containing either ten or fifty million organisms.

---

**"Tabloid" Chemicals for Colour Photography.**—Burroughs, Wellcome & Co., London.—The production of photographs in natural colours, apart from its artistic interest, is a valuable addition to the weapons of the scientist, and will doubtless become increasingly the recognised method of recording observations of pathological conditions wherever the element of colour is of importance. For instance, photographs of skin diseases, histological specimens and examples of abnormal growths gain much in clearness and in the amount of information conveyed, when faithfully reproduced in colour.

The chemical processes involved in the production of such photographs, for reversing the image and for intensifying the colours, have now been conveniently provided for in a set of three "Tabloid" products, which enable the amateur to experiment with all the brands of colour plates now on the market with the minimum expenditure of time and trouble, The whole process of development, reversal, re-development, and intensification can be carried out with solutions of "Tabloid," "Rytol," "Tabloid" Reversing Compound, and "Tabloid" Colour Plate Intensifier, and by following the instructions given, successful results in natural colour can be obtained with the Autochrome, Thames, Omnicolore, and Dufay plates.

---

**"Epinine."**—Burroughs, Wellcome & Co., London.—Since the introduction into therapeutics of the active principle of the suprarenal gland, now so widely used, many attempts have been made to produce a synthetic substance which should possess similar physiological properties, and at the same time be free from certain disadvantages of the natural principle. "Epinine," which chemically is 3.4-dihydroxyphenylethylmethylamine, was discovered at the "Wellcome" Chemical Works, and is stated to possess all the valuable physiological properties of the suprarenal principle, with some additional advantages. The most important of these latter are that it is much more stable than the suprarenal principle, and being a synthetic preparation, is more easily obtained in a state of purity. By physiological tests, "Epinine" has been proved to produce all the characteristic effects of the suprarenal principle on the blood-pressure, heart's action, uterus, etc. Quantitatively, it has been found that 1 in 100 solutions of "Epinine" are equal in activity to 1 in 1000 solutions of suprarenal active principle. The rise in blood-pressure produced by "Epinine" is, however, more prolonged than is the case with rises of equal height due to the suprarenal principle. "Epinine" is issued in the strength of 1 in 100 in bottles of 10 and 25 c.c., and also as a sterile solution in the convenient "Vaporole" containers.

*Tracings showing rise of blood pressure caused by "Epinine" and "Hemisine."*

A & C=0.5 mgm. 3.4 Dihydroxyphenylethylmethylamine ("Epinine").
B & D=0.05 mgm. Suprarenal active principle ("Hemisine").

*Note equal height of AC and BD, but greater persistence of AC.*

---

**"Vaporole" Ammonium Chloride Inhaler.**—BURROUGHS, WELLCOME & CO., London.—The number of Ammonium Chloride inhalers on the market proves the very real difficulties to be faced in producing a satisfactory pattern. The makers claim for this inhaler that it is compact, easily and quickly used, and that it provides a perfectly neutral vapour.

We think that this instrument fully justifies these claims. By the use of "Vaporole" sealed charges of acid and ammonia the inhaler can be set up in a moment; it is easily taken to pieces to clean after use, and the vapour produced is certainly not irritating.

In cases in which the inhalation of Ammonium Chloride Vapour is considered advisable we can recommend the use of this apparatus.

---

**"Vaporole" Hemisine and Cocaine.**—BURROUGHS, WELLCOME & Co., London.—The reliable anæsthetic effects with lessened toxicity which the more recent substitutes for cocaine possess has to a large extent limited the use of the latter drug for hypodermic injection. Cocaine, however, is still used to some extent, combined with extract of suprarenal gland for use in cases where a bloodless field of operation is particularly desired, and in such cases this preparation (containing 2 per cent. cocaine) will be found convenient, owing to its sterility and exact dosage.

The combination of "Hemisine" and Cocaine Hydrochloride is a useful preparation for dental practice. "Hemisine" is the well-known preparation of the suprarenal active principle, and it is here combined with cocaine in just the right proportion to enhance the value of the latter, without causing too much constriction of the capillary blood vessels of the socket. There is thus no liability to sloughing. Clinical reports show that the anæsthesia produced is very satisfactory.

Each c.c. contains "Hemisine" gr. $\frac{1}{2160}$ and Cocaine Hydrochloride gr. $\frac{1}{3}$.

*Direction.*—One c.c. to be injected into the gums for the production of local anæsthesia.

---

**Sophol.**—THE BAYER CO. LTD., London.—This preparation has been recently introduced as a substitute for silver nitrate in the treatment of ophthalmia neonatorum. It is said to be about three times as active a bactericide as protargol, and to cause less smarting and irritation, in which respect it resembles argyrol. We have used it in solutions of from 2 to 10 per cent. without noticing any very considerable irritation. Sophol is a formo-nucleinate of silver, containing 20 per cent. of the metal. It is a yellow powder, easily soluble in cold water. The solutions should be freshly prepared and carefully kept from the action of light. We have no experience of it as a prophylactic against ophthalmia neonatorum, but it is certainly a harmless and in many cases an efficient remedy in acute conjunctivitis, and deserves a trial wherever other silver compounds have proved ineffectual.

## Figures and Tables

**Figure f1:**